# A Breeding-Informed Regulatory Screen Identifies ZmSPL19 as a Negative Regulator of Nitrogen-Sufficient Growth in Maize (*Zea mays* L.)

**DOI:** 10.3390/plants15091387

**Published:** 2026-04-30

**Authors:** Zhijing Bai, Xinle Zhu, Changyu Li, Binbin Zhao, Lian Jin, Baobao Wang

**Affiliations:** 1 Biotechnology Research Institute, Chinese Academy of Agricultural Sciences, Beijing 100081, China; 15132820614@163.com (Z.B.); zhuxinle0903@163.com (X.Z.); nkylichangyu@163.com (C.L.); zhaobinbin@ahau.edu.cn (B.Z.); jla13453556961@163.com (L.J.); 2 The National Engineering Laboratory of Crop Resistance Breeding, School of Life Sciences, Anhui Agricultural University, Hefei 230036, China; 3 National Nanfan Research Institute (Sanya), Chinese Academy of Agricultural Sciences, Sanya 572025, China

**Keywords:** maize, ZmSPL19, transcription factor, nitrogen use efficiency, breeding-era transcriptome, nitrogen assimilation

## Abstract

Nitrogen use efficiency (NUE) is a major determinant of maize (*Zea mays* L.) productivity and sustainability, yet the regulatory changes associated with modern breeding remain incompletely understood. Here, we used breeding-era transcriptomic data from 137 elite Chinese maize inbred lines to identify transcriptional regulators associated with maize NUE. Breeding-era expression shifts in NUE effector genes were modest but tissue-specific, pointing to pathway-level transcriptional rewiring during modern breeding. Focusing on the first leaf above the uppermost ear at silking, we identified 69 breeding-era-responsive genes, including 10 transcription factors, and prioritized *ZmSPL19* through Pearson correlation analysis with curated NUE-related genes. *ZmSPL19* expression declined during modern breeding and showed a nitrate-repressed expression, with lower transcript abundance under nitrogen-sufficient conditions and rapid downregulation upon nitrate resupply. Loss of *ZmSPL19* function promoted primary root elongation, biomass accumulation, leaf nitrogen content, soil–plant analysis development (SPAD), photosynthetic rate, kernel number, and grain yield under nitrogen-sufficient conditions. These results identify ZmSPL19 as a breeding-associated negative regulator of growth and yield formation under nitrogen-sufficient conditions and support the value of a breeding-informed strategy for discovering regulators with potential relevance to maize NUE improvement.

## 1. Introduction

Maize (*Zea mays* L.) is one of the most important cereal crops worldwide and a major source of food, feed, and industrial raw materials [1,2]. Nitrogen (N) is indispensable for plant growth and productivity [3,4], but maize production has long depended on excessive N fertilizer input to sustain high yields [5]. This dependence comes at a substantial cost, as the low recovery and conversion efficiency of applied N in maize-based cropping systems not only reduces input efficiency but also increases the risks of soil acidification, nitrate loss, and water eutrophication [6,7,8]. Improving nitrogen use efficiency (NUE) in maize is therefore essential for sustaining grain production while reducing the environmental burden of intensive agriculture.

At the molecular level, recent studies have identified important regulators of N uptake, transport, assimilation, and signaling in maize. Nitrate transporters and their regulators contribute directly to N acquisition, as illustrated by *ZmNRT1.1B* and *ZmEREB97* [9,10]. Genes involved in N assimilation have also been reported, including *ZmGS1-3*/*4*, *THP9*, and *ZmFD4* [11,12,13]. In parallel, NIN-like proteins (NLPs) have emerged as key hubs in maize N signaling and metabolism. *ZmNLP5*, *ZmNLP3.2*, and *ZmNLP8* have all been linked to nitrate-responsive growth and nitrogen assimilation, highlighting the importance of transcriptional control in NUE-related traits [14,15,16]. Despite these advances, the regulatory changes associated with breeding-era improvement remain poorly characterized.

Breeding-era population resources provide a useful resource for addressing this question. A recent study characterized 137 elite Chinese maize inbred lines from different breeding eras and showed that multiple yield-related traits increased during modern breeding. Notably, this population has also generated transcriptomic gene expression data for key tissues [17]. Such resources make it possible to identify genes with expression changes during breeding. However, for a complex trait such as NUE, expression change alone is often insufficient to prioritize the most relevant candidates [18]. A more informative strategy is to interpret these changes in relation to biologically connected genes and pathways, to efficiently anchor candidate genes to components of NUE-related pathways [19,20]. We therefore focused on the first leaf above the uppermost ear at silking, a major source tissue for maize N metabolism, and prioritized breeding-era-responsive transcription factors by their association with curated NUE genes involved in uptake/transport/remobilization, reduction, and assimilation.

In this study, breeding-informed analysis identified 10 transcription factors with monotonic breeding-era-responsive expression patterns in the first leaf above the uppermost ear at silking, all of which showed lower expression in modern lines than in older lines. Among them, *ZmSPL19* encodes an SQUAMOSA PROMOTER BINDING LIKE (SPL) transcription factor [21,22] and was selected here as one candidate for proof-of-concept functional analysis because it combined a clear monotonic decline across breeding eras, a nitrogen-related association pattern in the ear-leaf transcriptome, nitrogen-responsive expression, and prior agronomic relevance of SPL-family genes in maize. We do not regard *ZmSPL19* as the only candidate identified by the screen; rather, it was prioritized here as one well-supported example for functional validation. Using CRISPR/Cas9-generated lines, we found that loss of *ZmSPL19* enhances root growth, biomass accumulation, leaf nitrogen status, photosynthetic performance, kernel number, and grain yield predominantly under nitrogen-sufficient conditions. Together, these results identify ZmSPL19 as a breeding-relevant negative regulator of growth and yield formation under nitrogen-sufficient conditions, with potential value as a target for improving maize NUE and productivity, and illustrate the utility of a breeding-informed framework for discovering actionable genes for NUE-oriented crop improvement.

## 2. Results

### 2.1. Breeding-Era Expression Shifts in Maize NUE Effector Genes Suggest Pathway-Level Transcriptional Rewiring

**Figure 1:**
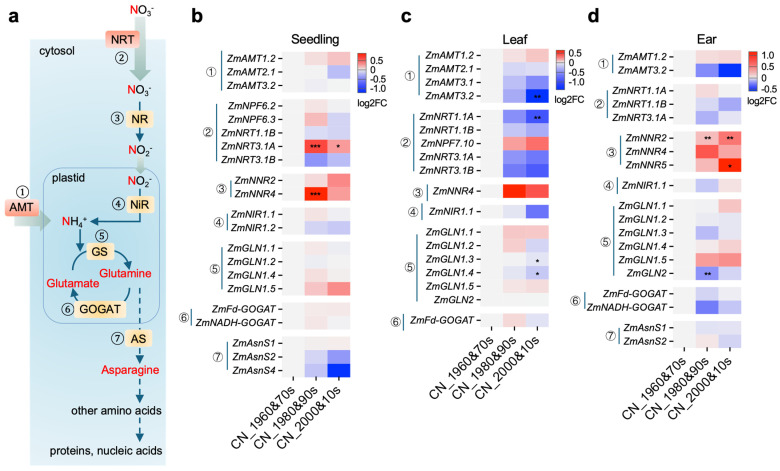
Expression divergence of nitrogen uptake and primary assimilation genes across Chinese maize (*Zea mays* L.) breeding eras. (**a**) Schematic of nitrate uptake and primary nitrogen assimilation in plants. Key proteins are numbered (①–⑦): ① ammonium transporter (AMT), ② nitrate transporter (NRT), ③ nitrate reductase (NR), ④ nitrite reductase (NiR), ⑤ glutamine synthetase (GS), ⑥ glutamate synthase (GOGAT), and ⑦ asparagine synthetase (AS). The same numbering is used in (**b**–**d**) to indicate the corresponding gene sets encoding these proteins. (**b**) Heatmap showing expression changes of nitrogen uptake/assimilation-related genes in seedlings at the V2 stage from 137 elite inbred lines of maize from different breeding eras in China. (**c**,**d**) Heatmaps showing expression changes at the silking stage, with the first leaf above the uppermost ear (**c**) and unpollinated ear (**d**). The analysis was performed for all genes listed in Table 1; however, only genes with available expression data in the corresponding dataset are shown in the heatmaps. In addition, to avoid unstable fold-change estimates caused by near-zero expression, genes were required to satisfy an expression floor of min(median_CN_1960&70s, median_CN_1980&90s, median_CN_2000&10s) ≥ 0.1; therefore, some genes listed in Table 1 are not displayed. For (**b**–**d**), heatmap values are log2 fold changes (log2FC) relative to CN_1960&70s, calculated from the median expression across inbred lines within each era. Pairwise comparisons were performed between each breeding era and the CN_1960&70s group. Homogeneity of variance was first assessed using an F-test. When variances were not significantly different (*p* > 0.05), a two-sided Student’s *t*-test was used; when variances were significantly different (*p* < 0.05), a two-sided Welch’s *t*-test was applied. All tests were conducted on untransformed FPKM values. Asterisks indicate significance levels (* *p* < 0.05, ** *p* < 0.01, *** *p* < 0.001).

Given the widespread use of synthetic nitrogen fertilizer and the long-term prevalence of relatively high nitrogen input in modern agriculture, we hypothesized that modern maize breeding may have been accompanied by transcriptional rewiring of nitrogen use efficiency (NUE)-related gene networks. To test this idea, we compiled genes involved in nitrogen uptake, transport, and assimilation (hereafter referred to as NUE effector genes) from published studies and summarized their expression across a breeding-era transcriptome dataset comprising 137 elite Chinese maize inbred lines. This dataset included three tissues and developmental contexts: seedling (V2), leaf (first leaf above the uppermost ear at silking), and ear (unpollinated ear at silking) (Table 1 and Figure 1a–d).

Across all three tissues, NUE effector genes showed breeding-era-associated expression shifts, but these changes were generally modest in magnitude (most |log2FC| values ≤ 2.5) and strongly tissue-specific. Even so, small but coordinated directional shifts were evident within functional groups. For example, nitrate reductase genes showed clear breeding-era upregulation in specific tissues, including *ZmNNR4* in seedlings and *ZmNNR2* and *ZmNNR5* in ears (Figure 1b,d). By contrast, in the first leaf above the uppermost ear at silking, genes related to nitrogen transport tended to be downregulated during breeding, with significant decreases observed for *ZmAMT3.2* and *ZmNRT1.1A*, and several assimilation-related genes also showed downward trends (Figure 1c). Thus, this pattern—distributed across multiple genes, modest in amplitude, and tissue-specific in manifestation—argued against a model driven by large expression changes in single effector genes. Instead, it suggested pathway-level transcriptional reconfiguration during modern breeding and raised the possibility that such reconfiguration was driven, at least in part, by regulatory factors [23].

### 2.2. Breeding-Era-Responsive Transcription Factors and Their Association Patterns with NUE Genes Prioritize ZmSPL19 for Proof-of-Concept Functional Analysis

**Figure 2:**
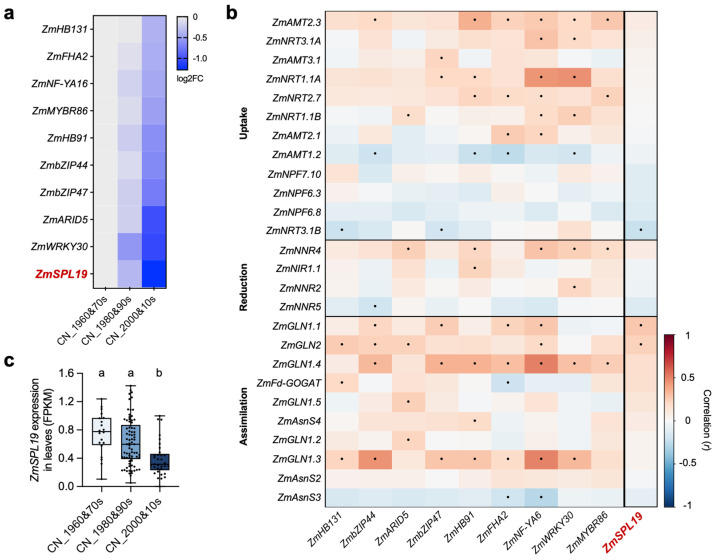
Breeding-era-responsive transcription factors and their association patterns with NUE genes in maize. (**a**) Heatmap showing expression changes in the 10 transcription factors (TFs) identified from breeding-era-responsive genes in the first leaf above the uppermost ear at silking across three Chinese maize breeding eras. Heatmap values are log2 fold changes (log2FC) relative to the CN_1960&70s group, calculated from the median expression across inbred lines within each era. (**b**) Pearson correlation heatmap showing the relationships between the 10 breeding-era-responsive TFs and nitrogen use efficiency (NUE)-related genes (in Table 1) across 137 maize inbred lines. NUE genes are grouped by function into uptake/transport/remobilization, reduction, and assimilation. Heatmap colors represent Pearson correlation coefficients (*r*). Dots indicate nominal significance at *p* < 0.05. (**c**) Expression levels (FPKM) of *ZmSPL19* in the first leaf above the uppermost ear at silking across breeding eras. Each dot represents one inbred line; boxplots show the median and interquartile range. Multiple comparisons were performed using the LSD test in the agricolae package (v0.1.16) in R (v4.5.2), with Bonferroni correction (*p* < 0.05). Different lowercase letters indicate significant differences among groups. In (**a**,**b**), *ZmSPL19* is highlighted in red to indicate the candidate selected for further functional analysis.

Because the leaf at silking is a major source tissue in maize nitrogen metabolism [24], we next focused on the leaf transcriptome to identify breeding-era-responsive transcription factors potentially associated with NUE-related rewiring. Using the revised and more stringent screening criteria (see Section 4), we identified 69 breeding-era-responsive genes in the first leaf above the uppermost ear at silking (Table S1). Functional annotation based on MaizeGDB indicated that 10 of these genes encode transcription factors (Table S1). All 10 TFs showed monotonic downregulation across breeding eras, with generally lower expression in modern lines than in older lines (Figure 2a). Among them, *ZmSPL19* showed a clear monotonic decline across breeding eras and the largest decrease between CN_2000&10s and CN_1960&70s (Figure 2a,c).

To further examine the NUE-related context of these TFs, we performed Pearson correlation analysis between the 10 breeding-era-responsive TFs and NUE-related genes (in Table 1) across the 137 maize inbred lines. The resulting association patterns differed among TFs and across functional modules. Notably, *ZmSPL19* (Zm00001d053775) displayed a module-biased association pattern, showing preferential positive correlations with several assimilation-related genes, but negative correlations with part of the uptake/transport module (Figure 2b). This analysis was used for candidate prioritization rather than mechanistic inference.

We next incorporated nitrogen-response profiling as an additional prioritization criterion. Comparative RT-qPCR analysis of the 10 candidate TFs during nitrate and ammonium resupply revealed diverse response patterns (Figure S1; Table S2). Among them, *ZmSPL19* displayed the clearest and most sustained repression after nitrate resupply, whereas *ZmWRKY30* showed a more transient decrease (Figure S1a,b; Table S2). Under ammonium resupply, the candidate TFs also showed heterogeneous responses, and *ZmSPL19* did not display the same sustained repression observed under nitrate resupply (Figure S1c,d; Table S2). These data suggest that nitrogen-response profiling provides an additional criterion for candidate prioritization and that *ZmSPL1*9 is distinguished by a nitrate-repressed, nitrogen-form-specific response pattern. Taken together, its clear monotonic decline across breeding eras, nitrogen-related association pattern, nitrate-repressed response profile, and prior agronomic relevance of SPL-family genes in maize led us to prioritize *ZmSPL19* as one well-supported candidate for functional analysis [21,25]. We note, however, that the screen identified multiple plausible TF candidates, and other TFs such as *ZmFHA2* and *ZmARID5* also remain of interest for future study.

### 2.3. ZmSPL19 Is Downregulated During Modern Breeding and Shows Nitrate-Repressed Expression

To further evaluate whether *ZmSPL19* is connected to breeding-related NUE regulation, we first examined its breeding-era expression trajectory. In the first leaf above the uppermost ear at silking, *ZmSPL19* was significantly downregulated from earlier to modern breeding eras, suggesting that its expression was weakened during modern breeding (Figure 2c). To further examine whether the genomic region around *ZmSPL19* showed evidence of breeding-associated changes, we analyzed genome-wide SNP data from the same 137 Chinese maize inbred lines and calculated nucleotide diversity (π) and Tajima’s D in 50 kb non-overlapping windows. In the 50 kb window overlapping the *ZmSPL19* gene body, π decreased from 0.00324 in CN_1960&70s to 0.00201 in CN_2000&10s, accompanied by a reduction in Tajima’s D from 1.69 to 0.32 (Table S3). The downstream flanking window also showed reduced π in modern lines and a negative Tajima’s D value in CN_2000&10s (Table S3). These results provide supportive evidence for a local reduction in genetic diversity around *ZmSPL19* during modern breeding, although they do not by themselves identify the causal variant underlying its expression change.

**Figure 3:**
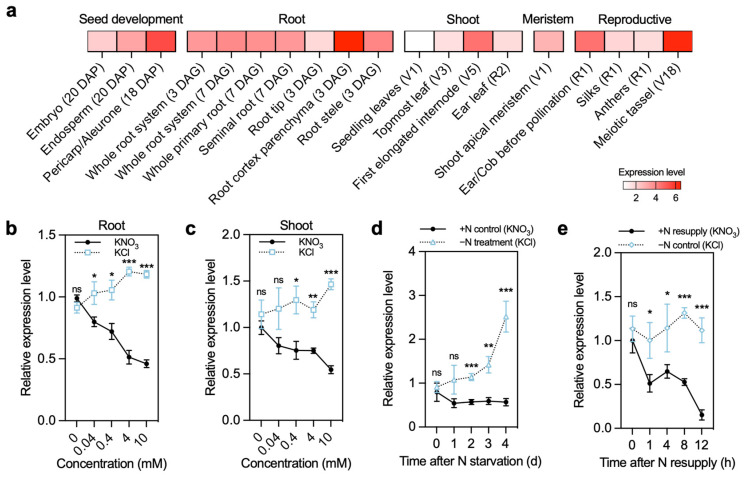
Transcriptional responses of *ZmSPL19* to varying nitrate availability and nitrogen starvation–resupply treatment. (**a**) Tissue-level expression pattern of Zm00001d053775 (*ZmSPL19*) in selected maize (B73) tissues from the MaizeGDB qTeller expression atlas. Tissue labels include developmental timing where applicable: DAP indicates days after pollination, and DAG indicates days after germination. The Root tip sample corresponds to the combined meristematic and elongation zones. Stage designations follow standard maize vegetative (V) and reproductive (R) staging. (**b**,**c**) Transcriptional responses of *ZmSPL19* to different nitrate concentrations in roots (**b**) and shoots (**c**) based on RT–qPCR. KCl was used at the same concentrations as KNO_3_ as a control to exclude a K^+^ effect. (**d**,**e**) Dynamic transcriptional responses of *ZmSPL19* under nitrogen starvation (**d**) and nitrogen resupply (**e**). Maize (B73) seedlings were grown in 4 mM KNO_3_ for 5 d, transferred to N-free nutrient solution (4 mM KCl was supplemented to maintain consistent K^+^ concentration and osmotic potential) for 1, 2, 3, and 4 d, followed by resupply of 4 mM KNO_3_ for 1, 4, 8, and 12 h. In (**b**–**e**), data are means ± s.d. derived from *n* = 3 biological replicates (three plants per replicate). * *p* < 0.05, ** *p* < 0.01, *** *p* < 0.001, ns *p* > 0.05 compared with control, Student’s *t*-test.

We next examined its expression pattern across tissues and developmental stages and found that *ZmSPL19* is constitutively expressed across multiple organs, indicating that it is not restricted to a single developmental compartment (Figure 3a). Because maize is typically cultivated as an upland crop and nitrate represents a major inorganic nitrogen source in well-aerated soils [26,27], we next focused on the nitrate responsiveness of *ZmSPL19* in greater detail. In seedling nitrate-gradient experiments, *ZmSPL19* transcript abundance decreased progressively with increasing external KNO_3_ concentration in both roots and shoots, indicating a dose-dependent negative response to nitrogen supply (Figure 3b,c). We further tested its dynamic response using nitrogen starvation and nitrate resupply assays. Under nitrogen deprivation, *ZmSPL19* expression increased over time, becoming significantly elevated by day 2 and reaching nearly 2.5-fold by day 4 (Figure 3d). By contrast, upon nitrate resupply (4 mM KNO_3_), *ZmSPL19* expression dropped rapidly within 1 h, followed by a slight transient rebound, yet remained at lower levels by 12 h (Figure 3e), indicating a clear nitrate-repressed response pattern. A supplementary NH_4_Cl resupply assay further showed that *ZmSPL19* also responded to ammonium, but with a weaker and qualitatively different pattern from that observed under nitrate resupply (Figure S1c,d; Table S2). Together, these results indicate that *ZmSPL19* is more characteristically repressed by nitrate and support its classification as a nitrate-repressed, nitrogen-form-sensitive transcription factor.

### 2.4. Generation of Zmspl19 Lines and Seedling Growth Phenotypes Under Different Nitrogen Conditions

**Figure 4:**
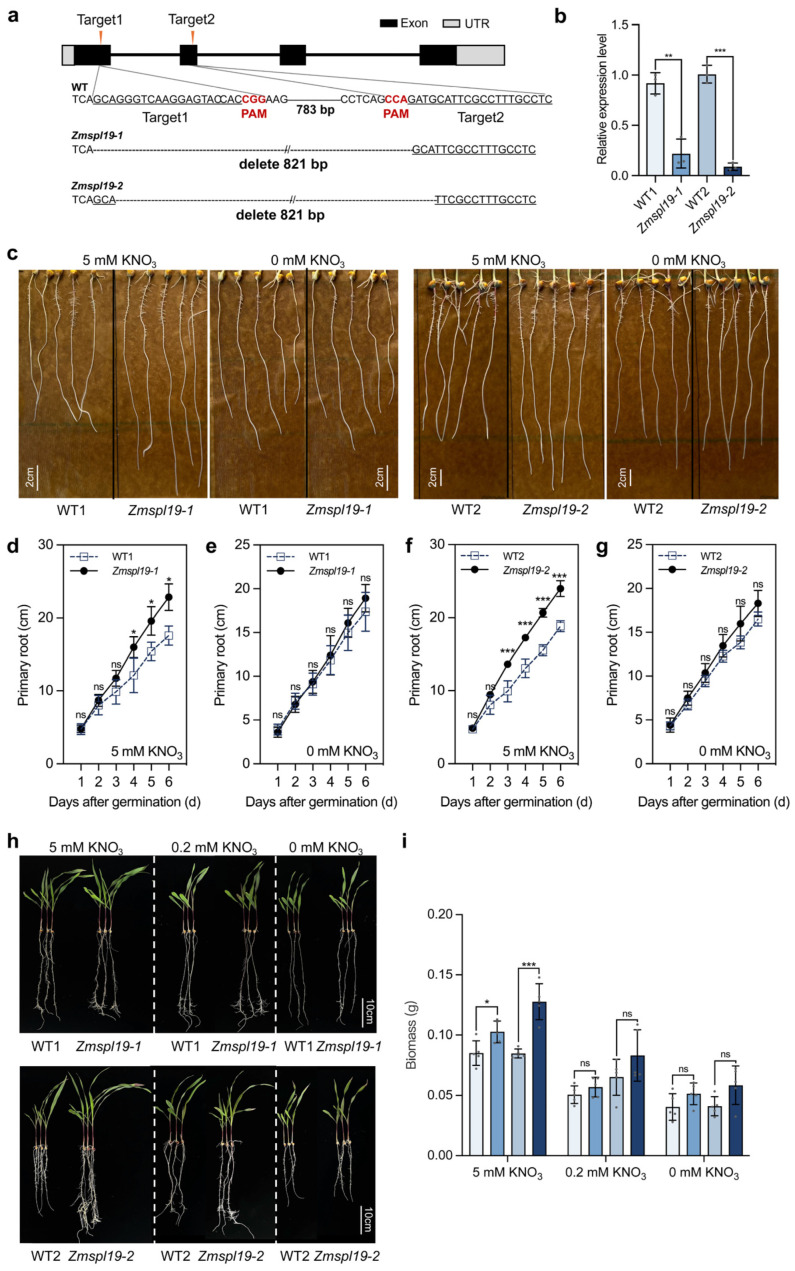
*ZmSPL19* loss of function affects nitrate-dependent growth. (**a**) Schematic of CRISPR/Cas9-induced loss-of-function lines in *ZmSPL19*. Two guide RNA target sites (Target1 and Target2) were designed within the *ZmSPL19* genomic region (exons shown in black; UTRs in gray). The wild-type (WT) sequence is shown, with the protospacer adjacent motif (PAM) sites indicated. Two independent edited alleles, *Zmspl19-1* and *Zmspl19-2*, carry a large deletion between Target1 and Target2 (821 bp), generated by CRISPR/Cas9-mediated double-strand breaks and subsequent repair. (**b**) Relative expression of *ZmSPL19* in V2 shoots of two independent loss-of-function lines and their corresponding wild-type controls. *n* = 3 biological replicates (three plants per replicate). (**c**) Representative images of primary roots of WT1/*Zmspl19-1* and WT2/*Zmspl19-2* seedlings grown under 5 mM KNO_3_ or 0 mM KNO_3_. Nitrate treatments were applied immediately after germination; photographs were taken on day 6 of treatment. Scale bars, 2 cm. (**d**–**g**) Primary root length was recorded daily over a 6-day treatment period under 5 mM KNO_3_ (**d**,**f**) or 0 mM KNO_3_ (**e**,**g**). WT1 vs. *Zmspl19-1* are shown in (**d**,**e**), and WT2 vs. *Zmspl19-2* are shown in (**f**,**g**) (*n* = 5 plants). (**h**) Seedling phenotypes of wild-type KN5585 (WT1 and WT2) and *Zmspl19* mutant (*Zmspl19-1* and *Zmspl19-2*) plants grown under different nitrate supplies (5 mM KNO_3_, 0.2 mM KNO_3_, and 0 mM KNO_3_) for two weeks. Scale bars, 10 cm. (**i**) Biomass of wild-type KN5585 and *Zmspl19* mutant seedlings under different nitrate supplies (*n* = 5 plants). In (**b**,**d**–**g**,**i**), data are presented as mean ± s.d., and * *p* < 0.05, ** *p* < 0.01, *** *p* < 0.001, and ns *p* > 0.05 compared with wild type, Student’s *t*-test.

To test whether *ZmSPL19* restricts yield formation under agronomically relevant nitrogen supply, we generated CRISPR/Cas9 loss-of-function lines in the maize inbred line KN5585. Two independent edited events were obtained, each predicted to introduce a premature stop codon and disrupt the SBP DNA-binding domain (Figure 4a). In both mutant lines, *ZmSPL19* transcript abundance was significantly reduced relative to their corresponding wild-type controls (Figure 4b).

To assess how *ZmSPL19* influences nitrogen-related growth, we first examined seedling root growth under contrasting nitrogen conditions. Under nitrogen-free conditions, primary root length did not differ significantly between *Zmspl19-1*, *Zmspl19-2*, and their corresponding wild-type controls (WT1 and WT2) (Figure 4c,e,g). Under nitrogen-supplied conditions, however, both mutants developed significantly longer primary roots from 4 to 6 d after germination (Figure 4c,d,f), indicating that loss of *ZmSPL19* promotes post-germination root growth specifically in the presence of nitrogen.

This nitrogen-dependent effect was also reflected in biomass accumulation. Under nitrogen-free and low-nitrogen conditions, total biomass did not differ significantly between mutants and their matched controls. Under high-nitrogen conditions, by contrast, both *Zmspl19-1* and *Zmspl19-2* accumulated significantly more biomass than their corresponding wild types, with increases of 20.1% and 50.6%, respectively (Figure 4h,i). Thus, the growth-promoting effect of *ZmSPL19* loss of function is primarily expressed under nitrogen-sufficient conditions.

### 2.5. Loss of ZmSPL19 Enhances Mature-Stage Performance, Ear-Leaf Physiological and Transcriptional Traits Under Nitrogen-Sufficient Conditions

**Figure 5:**
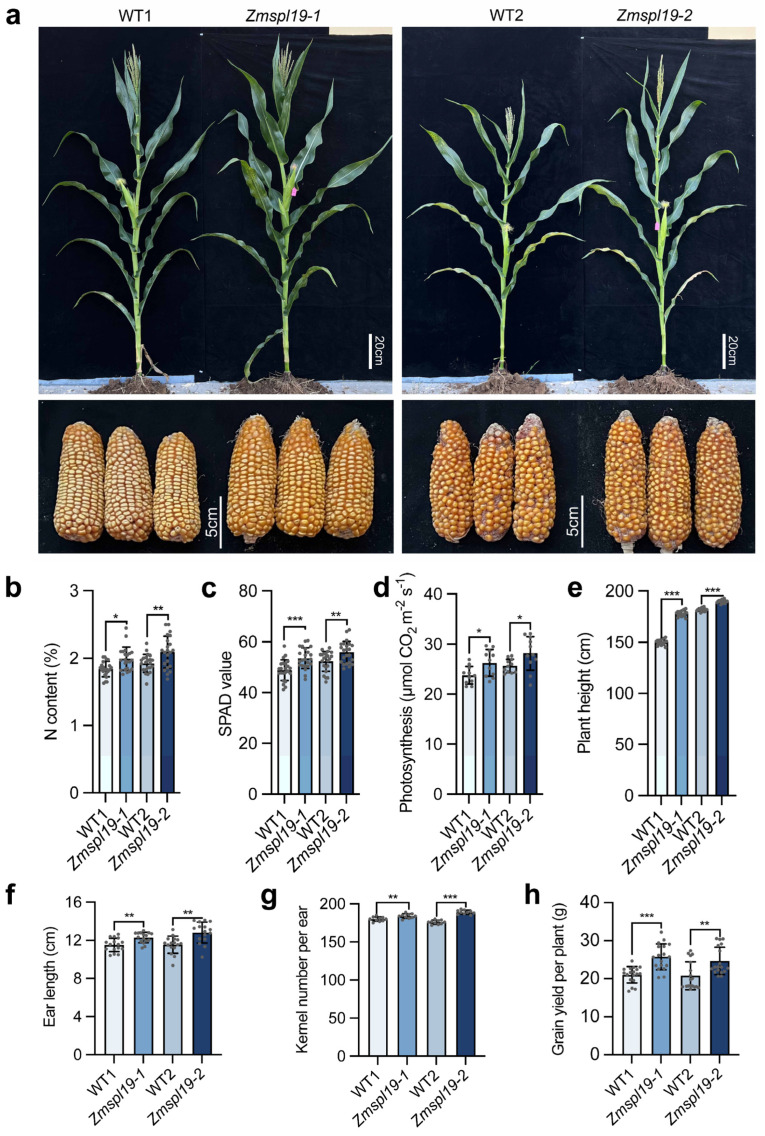
*ZmSPL19* loss of function affects physiological traits and grain yield under nitrogen-sufficient conditions. (**a**) Shoot (**top**: Scale bars, 20 cm) and ear (**bottom**: Scale bars, 5 cm) phenotypes for mature wild-type KN5585 (WT1 and WT2) and *ZmSPL19* knockout (*Zmspl19-1* and *Zmspl19-2*) plants grown under normal nitrogen conditions (200 kg N ha^−1^) and harvested in Sanya, China, in 2023. (**b**–**d**) Ear leaf N content ((**b**), *n* = 22 plants), ear leaf SPAD value ((**c**), *n* = 23 plants), and ear leaf net photosynthesis rate ((**d**), *n* = 11 plants) for mature wild-type KN5585 and *Zmspl19* mutant plants grown under normal nitrogen conditions (200 kg N ha^−1^). For net photosynthesis rate, data were collected under field conditions with an air temperature of 30 °C, a CO_2_ concentration of 400 ppm, and a fixed light intensity of 1800 µmol photons m^−2^ s^−1^. (**e**–**h**) Plant height ((**e**), *n* = 16 plants), ear length ((**f**), *n* = 16 plants), kernel number per ear ((**g**), *n* = 10 plants), and grain yield per plant ((**h**), *n* = 18 plants) for wild type and two *Zmspl19* mutant lines. The wild-type corresponding to each mutant line is derived from the segregating progeny of the same transgenic event. In (**b**–**h**), data are presented as mean ± s.d., and * *p* < 0.05, ** *p* < 0.01, and *** *p* < 0.001 compared with wild type, Student’s *t*-test.

Having observed that loss of *ZmSPL19* promoted seedling growth mainly under nitrogen-supplied conditions, we next examined whether this effect was reflected in mature plants grown under nitrogen-sufficient field conditions. Field evaluation was conducted in Sanya, Hainan, China, using a planting density of 60,000 plants ha^−1^ and a total nitrogen input of 200 kg N ha^−1^, representing a relatively high-nitrogen production setting. At maturity, both loss-of-function lines also showed a significant increase in plant height (Figure 5e), consistent with the possibility that ZmSPL19 shares growth-regulatory features with its close homolog ZmSPL12 [25]. In addition, both *Zmspl19-1* and *Zmspl19-2* showed significant increases in ear length and kernel number (Figure 5a,f,g). Ear length increased by 6.7% in *Zmspl19-1* and 11.1% in *Zmspl19-2*, whereas kernel number increased by 2.2% and 7.4%, respectively. Consistent with these improvements in ear traits, grain yield per plant was significantly higher in both mutants, with increases of 4.7 g per plant in *Zmspl19-1* and 3.8 g per plant in *Zmspl19-2* relative to their matched controls (Figure 5h). Together, these results indicate that loss of *ZmSPL19* is associated with increased grain yield under nitrogen-sufficient conditions.

We further examined whether these mature-stage phenotypes were associated with altered nitrogen-related physiology under the same nitrogen-sufficient field conditions. In the ear leaf, both mutants showed significantly higher leaf nitrogen content, SPAD value, and net photosynthetic rate than their corresponding controls (Figure 5b–d). Net photosynthetic rate increased by 10.4% in *Zmspl19-1* and 9.8% in *Zmspl19-2* (Figure 5d).

**Figure 6:**
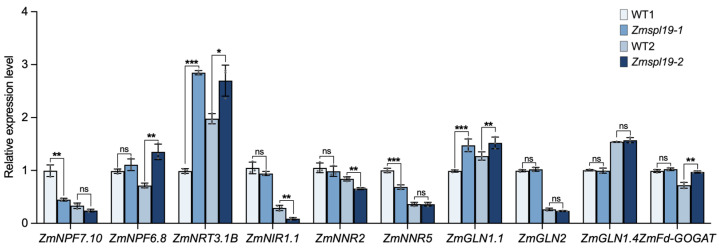
Expression analysis of selected NUE-related genes in ear leaves of *Zmspl19* mutants under nitrogen-sufficient field conditions. Relative transcript abundance of the indicated NUE-related genes was determined by RT-qPCR in the middle region of the ear leaf at the silking stage from field-grown WT1, *Zmspl19-1*, WT2, and *Zmspl19-2* plants grown under nitrogen-sufficient conditions (200 kg N ha^−1^). Relative expression values are presented as 2^−ΔΔCt^, using WT1 as the common calibrator (WT1 = 1). Bars represent means ± s.d. of three biological replicates. Statistical significance was evaluated between each mutant and its matched wild type. * *p* < 0.05, ** *p* < 0.01, *** *p* < 0.001; ns, not significant.

To examine whether loss of *ZmSPL19* altered the nitrogen-related transcriptional state in mature leaves, we performed RT-qPCR analysis of NUE-related genes selected from the uptake/transport, reduction, and assimilation modules based on their Pearson correlation ranking with *ZmSPL19* in Figure 2b, using the top four genes from each module as initial candidates. Among these selected genes, *ZmNPF6.3* and *ZmNNR4* showed extremely low transcript abundance in nitrogen-sufficient ear leaves (Ct values close to 40) and were therefore excluded from quantitative interpretation. For the remaining genes, the effects of *ZmSPL19* loss were heterogeneous and gene-dependent rather than uniformly directional (Figure 6). Notably, *ZmNRT3.1B* and *ZmGLN1.1* showed increased expression in both mutant backgrounds, whereas most of the other tested genes displayed either line-dependent or statistically non-significant changes. Thus, these data provide limited but supportive evidence that loss of *ZmSPL19* is associated with changes in the nitrogen-related transcriptional state of ear leaves under nitrogen-sufficient conditions, while arguing against a simple uniform effect on the tested NUE genes.

Together, these results indicate that loss of *ZmSPL19* enhances mature-stage performance under nitrogen-sufficient conditions and is associated with coordinated changes in ear-leaf physiological traits and the nitrogen-related transcriptional state.

## 3. Discussion

In this study, by analyzing gene expression patterns across different breeding eras, we identified that genes associated with nitrogen use efficiency (NUE) may undergo expression rewiring during modern maize breeding. Several genes, including *ZmAMT1.2*, *ZmNNR2*, *ZmNNR4* and *ZmGLN1.5* (Figure 1), displayed a consistent upward expression trend in different tissues across breeding eras, implying that these genes may serve as vital contributors to the genetic improvement in modern maize breeding. Furthermore, among the NUE effector genes, despite differences in specific gene identities, genes involved in nitrogen uptake or transport predominantly exhibited a downward expression trend over breeding eras (Figure 1), especially in the first leaf above the uppermost ear at silking. It is well known that modern maize has long been cultivated under conditions of excessive nitrogen fertilizer application [28,29]. Such agronomic practices might enable maize to maintain adequate nitrogen availability in planta without relying heavily on robust nitrogen uptake and transport capacities [30,31]. The reduced expression of these nitrogen uptake- and transport-related genes may corroborate this inference. Therefore, the uptake/transport-related genes or processes that appear to have been relatively weakened during breeding may represent potentially promising targets for future improvement in maize NUE under reduced N input.

Benefiting from the breeding-era-assisted analysis, *ZmSPL19* was proved as a candidate NUE-related gene. The co-expressed NUE-related gene partners may provide insights into the potential mechanisms of its action. In the ear-leaf Pearson correlation network, ZmSPL19 showed only a limited number of nominally significant correlations with known NUE genes, but these correlations suggested a weak bias toward positive association with assimilation-related genes and negative association with part of the uptake/transport module (Figure 2b). This pattern is not inconsistent with its negative effect on nitrogen-sufficient growth, because co-expression reflects shared regulatory context across genotypes rather than the direction of direct regulation [32]. Together with its repression under nitrate supply and its stronger mutant phenotype under high N than under low or zero N (Figure 3 and Figure 4), these results suggest that ZmSPL19 may act upstream of a high-N leaf regulatory state that links nitrogen status with source activity and growth output. Consistent with this view, only *ZmNRT3.1B* and *ZmGLN1.1* showed reproducible increases in both mutant backgrounds, suggesting that the effect of *ZmSPL19* on N-related regulation is likely indirect rather than reflecting simple direct control of a canonical NUE pathway (Figure 6). Notably, the expanded nitrogen-response profiling of the 10 candidate TFs showed that their responses to nitrate and ammonium were not uniform. In this comparison, the FHA-domain TF *ZmFHA2* and the ARID-family TF *ZmARID5* showed particularly strong responses to nitrate and ammonium resupply, respectively (Figure S1), suggesting that the breeding-informed screen likely captured multiple nitrogen-responsive regulators with distinct preferences for different nitrogen forms. In this view, ZmSPL19 is less likely to determine a single uptake, transport, assimilation, or remobilization step directly, and more likely to modulate how these processes are coordinately deployed under nitrogen-sufficient conditions.

From a breeding perspective, *ZmSPL19* showed a continuous decline in expression level across breeding eras (Figure 2). Consistent with this pattern, local population-genetic analysis revealed reduced nucleotide diversity around the *ZmSPL19* gene body and downstream regions in modern breeding lines, together with lower Tajima’s D values (Table S3). These results support a possible breeding-associated reduction in variation around the *ZmSPL19* locus, although haplotype-resolved analyses will be needed to identify the causal variant(s) and test whether low-expression alleles were directly favored during breeding. The improved growth and yield-related traits of *Zmspl19* lines under nitrogen-sufficient conditions suggest that targeted reduction in ZmSPL19 activity may be useful for future breeding-oriented evaluation, potentially through genome-editing platforms such as IMGE [33]. However, the current field evaluation was conducted in a single year and a single environment, and plant architecture and yield performance under low-nitrogen field conditions were not assessed. Therefore, multi-environment, multi-year, and nitrogen-regime-specific evaluations will be needed before the breeding utility of *ZmSPL19* manipulation can be fully determined.

## 4. Materials and Methods

### 4.1. Identification of Breeding-Era-Responsive Transcription Factors and Population-Genetic Analysis of the ZmSPL19 Region

Publicly available expression data from the first leaf above the uppermost ear at silking, generated for 137 Chinese maize inbred lines in a previous study, were retrieved and reanalyzed here [17]. The lines had already been classified in that study into three breeding eras: CN_1960&70s (*n* = 21), CN_1980&90s (*n* = 82), and CN_2000&10s (*n* = 34). For each gene, the median expression value was calculated within each era, and only genes showing a strict monotonic trend across eras were retained (CN_2000&10s > CN_1980&90s > CN_1960&70s or CN_2000&10s < CN_1980&90s < CN_1960&70s). To avoid unstable fold-change patterns caused by near-zero expression, genes were additionally required to satisfy an expression floor of min(median_CN_1960&70s, median_CN_1980&90s, median_CN_2000&10s) ≥ 0.1. Pairwise comparisons were then performed using untransformed FPKM values, with CN_1960&70s as the reference group. For heatmap visualization, log2FC values were calculated relative to CN_1960&70s. For candidate-gene retention; however, breeding-era-responsive genes were required to show a strict monotonic trend and significant differences between CN_2000&10s and each of the two earlier breeding-era groups. Homogeneity of variance was first assessed using an F-test. When variances were not significantly different (*p* > 0.05), a two-sided Student’s *t*-test was used; when variances were significantly different (*p* < 0.05), a two-sided Welch’s *t*-test was applied. Breeding-era-responsive genes were defined as genes for which both CN_1960&70s versus CN_2000&10s and CN_1980&90s versus CN_2000&10s were significant at *p* < 0.001.

To examine whether the *ZmSPL19* region showed evidence of breeding-associated selection, genome-wide SNP data from the same 137 Chinese maize inbred lines were analyzed. The lines were grouped according to breeding era as CN_1960&70s, CN_1980&90s, and CN_2000&10s. Nucleotide diversity (π) and Tajima’s D were calculated separately for each group using VCFtools (v0.1.16) in 50 kb non-overlapping windows across the genome. *ZmSPL19* is located on chromosome 4 at 244,152,554–244,189,700 bp based on the B73 RefGen_v5 genome assembly. Three 50 kb windows spanning the *ZmSPL19* ± 50 kb region were extracted from the genome-wide results, corresponding to the upstream flanking region, the window overlapping the gene body, and the downstream flanking region. These window-based statistics were used to assess local changes in genetic diversity and allele-frequency patterns around *ZmSPL19* during modern maize breeding.

### 4.2. Pearson Correlation Analysis with NUE-Related Genes

Functional annotation of the resulting breeding-era-responsive genes was performed based on MaizeGDB, and genes annotated as transcription factors were retained for downstream analysis. To evaluate their association with nitrogen use efficiency, Pearson correlation analysis was performed between the expression levels of the breeding-era-responsive transcription factors and curated NUE-related genes (in Table 1) across the 137 maize inbred lines using the silking-stage ear-leaf dataset. Pearson correlation coefficients (*r*) and corresponding *p* values were calculated in R, and the resulting association matrix was visualized as a heatmap. Nominally significant correlations were defined at *p* < 0.05.

### 4.3. Nitrogen-Response Analysis of ZmSPL19

To examine the transcriptional response of *ZmSPL19* to nitrogen, maize inbred line B73 was used for hydroponic culture. Surface-sterilized seeds were germinated, and seedlings with radicles of approximately 1 cm were transferred to hydroponic culture and grown in water for 6 d under a 16 h light/8 h dark cycle at 25 °C until the one-leaf stage.

For nitrate-gradient treatment, seedlings were transferred to modified Hoagland solution containing KNO_3_ as the sole nitrogen source at 0, 0.04, 0.4, 4, or 10 mM. To control for potassium, parallel solutions containing equivalent concentrations of KCl instead of KNO_3_ were used. The basal solution contained 5 mM CaCl_2_, 2 mM MgSO_4_, 0.05 mM EDTA-Fe-Na, 0.5 mM KH_2_PO_4_, 50 μM H_3_BO_4_, 10 μM MnCl_2_, 1 μM ZnSO_4_, 0.3 μM CuSO_4_, and 0.5 μM Na_2_MoO_4_ (pH 5.8). The nutrient solution was renewed every 3 d. After 2 weeks of treatment, shoots and roots were harvested separately for expression analysis.

For nitrogen starvation and resupply experiments, seedlings were first grown for 5 d in Hoagland solution containing 4 mM KNO_3_, then transferred either to nitrogen-free solution in which 4 mM KNO_3_ was replaced by 4 mM KCl or maintained in 4 mM KNO_3_ as controls. Root samples were collected at 0, 1, 2, 3, and 4 d after nitrogen withdrawal. After 4 d of starvation, seedlings were transferred back to 4 mM KNO_3_ for nitrogen resupply, and root samples were collected at 0, 1, 4, 8, and 12 h.

For ammonium resupply experiments, the assay was performed with reference to a previously reported nitrogen-resupply approach [9]. B73 seedlings were hydroponically pre-cultured in nutrient solution containing 2 mM NH_4_NO_3_ for 10 d and then transferred to N-free nutrient solution for 96 h. After the starvation treatment, nitrogen was resupplied in the form of 4 mM NH_4_Cl, and samples were collected at 0, 1, 3, 6, and 12 h after resupply for RT-qPCR analysis. Whole maize seedlings at the V2 stage, defined as seedlings with the second leaf fully expanded and a visible, fully developed leaf collar, including both roots and shoots, were harvested for analysis.

### 4.4. Generation of Zmspl19 Loss-of-Function Lines

CRISPR/Cas9-mediated mutagenesis was used to generate *Zmspl19* mutant lines in the maize inbred background KN5585. Two guide RNAs targeting the coding region of *ZmSPL19* were designed using CRISPR-P 2.0 and assembled into the *pCPB-Ubi::hspCas9* vector as previously described [34]. The resulting construct was introduced into immature embryos by Agrobacterium tumefaciens-mediated transformation. Transgenic plants were screened by PCR, and editing events were verified by Sanger sequencing. Two independent homozygous loss-of-function lines, designated *Zmspl19-1* and *Zmspl19-2*, were identified and used for subsequent analyses. The matched wild-type controls WT1 and WT2 were derived from the segregating progeny of the corresponding transformation events. They were non-edited sibling plants and were confirmed to be free of the transgenic construct. These matched controls were used for comparisons with *Zmspl19-1* and *Zmspl19-2*, respectively.

### 4.5. Field Evaluation and Physiological Measurements Under Nitrogen-Sufficient Conditions

Field experiments were conducted in winter 2023 at the Yazhou Experimental Station, Sanya, Hainan, China (18.73° N, 109.17° E), using wild-type plants and the two independent loss-of-function lines *Zmspl19-1* and *Zmspl19-2*. Plants were grown at a density of 60,000 plants ha^−1^, consistent with local agronomic practice. Nitrogen-sufficient conditions were established using a split-fertilization regime. At the V4 stage (four-leaf stage), an initial fertilizer application was applied by furrow placement during intertillage to support early vegetative growth, including urea at 218 kg ha^−1^ (equivalent to 100 kg N ha^−1^), calcium superphosphate at 750 kg ha^−1^, and potassium chloride at 135 kg ha^−1^. A second urea application was then performed at the V12 stage (twelve-leaf stage), when nitrogen demand increases markedly during rapid vegetative development, at 218 kg ha^−1^ (equivalent to 100 kg N ha^−1^).

At silking, leaf nitrogen content was measured in the ear leaf using an N-Pen N110 nitrogen meter (Photon Systems Instruments, Drásov, Czech Republic). Measurements were taken at the basal, middle, and apical regions of the leaf, and the mean value was used for each plant. Relative chlorophyll content (SPAD value) was determined at the middle region of the ear leaf using an SPAD-502 meter (Konica Minolta, Inc., Sakai, Osaka, Japan), with five technical measurements averaged per sample. The net photosynthetic rate of the ear leaf was measured at silking using an LI-6400XT portable photosynthesis system (LI-COR Biosciences, Lincoln, NE, USA) between 10:00 and 12:00. Measurements were taken from the middle portion of the ear leaf under a photosynthetic photon flux density of 1800 μmol m^−2^ s^−1^, reference CO_2_ concentration of 400 ppm, flow rate of 500 μmol s^−1^, and ambient leaf chamber temperature of approximately 30 °C. For each plant, three technical measurements were recorded and averaged after signal stabilization.

Plants were open-pollinated and harvested 45 d after pollination. Ear length, kernel number per ear, and grain yield per plant, calculated as dried grain weight per plant, were recorded for phenotypic evaluation.

For additional RT-qPCR analysis, middle sections of ear leaves were collected from field-grown WT1, *Zmspl19-1*, WT2, and *Zmspl19-2* plants at approximately 10 d after silking under nitrogen-sufficient conditions (200 kg N ha^−1^). The selected NUE-related genes were chosen according to their Pearson correlation ranking with *ZmSPL19* in Figure 2b, and transcript abundance was analyzed as described in Section 4.7.

### 4.6. Primary Root Growth and Seedling Biomass Under Different Nitrogen Conditions

Wild-type, *Zmspl19-1*, and *Zmspl19-2* seeds were surface-sterilized, imbibed, and germinated on filter paper. For early primary root growth analysis, uniformly germinated seeds with radicles of 4–5 cm were transferred to Petri dishes containing moist filter paper supplied either with sterile water containing 5 mM KNO_3_ (+N) or with nitrogen-free solution in which 5 mM KCl was used to maintain potassium concentration (−N). For each genotype and treatment, five uniformly germinated seedlings were selected for primary root-length measurements, and individual seedlings were used as the statistical units (*n* = 5 plants). Seedlings were grown at 25 °C under a 16 h light/8 h dark cycle. Primary root length was recorded daily from digital images and quantified using ImageJ (v1.52k).

For seedling growth under graded nitrogen supply, germinated seeds with radicles of approximately 1 cm were transferred to a paper-roll hydroponic system. Seeds were arranged on moist germination paper, covered with a second sheet, rolled, and placed vertically in 2 L glass beakers containing modified Hoagland solution with high nitrogen (5 mM KNO_3_), low nitrogen (0.2 mM KNO_3_), or no nitrogen (0 mM KNO_3_); KCl was added as needed to equalize potassium among treatments. Rolls were grown at 25 °C under a 16 h light/8 h dark cycle, and the nutrient solution was renewed every 2 d. After 14 d, seedlings were harvested, rinsed with distilled water, oven-dried at 105 °C for 30 min and then at 80 °C to constant weight, and total dry biomass was determined.

### 4.7. RNA Extraction, cDNA Synthesis, and RT–qPCR Analysis

Collected samples were immediately frozen in liquid nitrogen and stored at −80 °C. Total RNA was isolated with TRIzol reagent (Invitrogen, Thermo Fisher Scientific, Waltham, MA, USA) following the manufacturer’s instructions. First-strand cDNA was synthesized using Hifair III 1st Strand cDNA Synthesis SuperMix for qPCR (gDNA digester plus) (YEASEN Biotech, Shanghai, China). Quantitative RT–PCR was carried out with Hieff UNICON qPCR SYBR Green Master Mix (YEASEN Biotech) on a Roche LightCycler 480 system (Roche Diagnostics GmbH, Mannheim, Germany). Three biological replicates were included for each assay. Relative transcript abundance was normalized to *Tubulin5* (Zm00001eb107490) and calculated using the 2^−ΔΔCt^ method. Primer sequences are provided in Table S4.

## Figures and Tables

**Table 1:** List of genes related to nitrogen absorption and assimilation in maize.

Encoding Protein Function	Gene	Gene ID_v3	Gene ID_v4
Ammonium transporter	*ZmAMT1.1*	GRMZM2G062024	-
	*ZmAMT1.2*	GRMZM2G164743	Zm00001d025894
	*ZmAMT1.3*	GRMZM2G028736	Zm00001d017249
	*ZmAMT2.1*	GRMZM2G080045	Zm00001d038412
	*ZmAMT3.1*	GRMZM2G335218	Zm00001d012261
	*ZmAMT3.2*	GRMZM2G338809	Zm00001d034782
	*ZmAMT3.3*	GRMZM2G043193	Zm00001d016771
Nitrate transporter	*ZmNPF6.2*	GRMZM2G064091	Zm00001d044529
	*ZmNPF6.3*	GRMZM2G476069	Zm00001d009399
	*ZmNPF6.4*/*ZmNRT1.1A*	GRMZM2G086496	Zm00001d024587
	*ZmNPF6.6*/*ZmNRT1.1B*	GRMZM2G161459	Zm00001d029932
	*ZmNRT1.1C*	GRMZM2G112154	-
	*ZmNRT1.1D*	GRMZM2G161483	Zm00001d027285
	*ZmNPF6.8*	GRMZM2G176253	Zm00001d016982
	*ZmNPF7.10*	GRMZM2G044851	Zm00001d017666
	*ZmNRT2.1*	GRMZM2G010280	Zm00001d054057
	*ZmNRT2.2*	GRMZM2G010251	Zm00001d054060
	*ZmNRT2.5*	GRMZM2G455124	Zm00001d011679
	*ZmNRT2.7*	-	Zm00001d044504
	*ZmNRT3.1A*	GRMZM2G179294	Zm00001d017095
	*ZmNRT3.1B*	GRMZM2G163494	Zm00001d003287
	*ZmNRT3*	GRMZM2G163866	Zm00001d014976
Nitrate reductase	*ZmNIAa*/*NNR5*	GRMZM2G428027	Zm00001d052139
	*ZmNIAb*/*ZmNNR2*	GRMZM5G878558	Zm00001d018206
	*ZmNIAc*/*ZmNNR1*	GRMZM2G568636	Zm00001d049995
	*ZmNIAd*/*ZmNNR4*	GRMZM2G076723	Zm00001d031769
Nitrite reductase	*ZmNIR1.1*	GRMZM2G079381	Zm00001d052164
	*ZmNIR1.2*	GRMZM2G102959	Zm00001d018161
Glutamine synthetase	*ZmGLN1.1*/*ZmGLN6*	GRMZM2G050514	Zm00001d028260
	*ZmGLN1.2*	GRMZM2G024104	Zm00001d033747
	*ZmGLN1.3*	GRMZM5G872068	Zm00001d017958
	*ZmGLN1.4*	GRMZM2G036464	Zm00001d051804
	*ZmGLN1.5*	GRMZM2G046601	Zm00001d048050
	*ZmGLN2*	GRMZM2G098290	Zm00001d026501
Glutamate synthase	*ZmFd-GOGAT*	GRMZM2G036609	Zm00001d022388
	*ZmNADH-GOGAT*	GRMZM2G077054	Zm00001d011610
Asparagine synthetase	*ZmAsnS1*	GRMZM2G074589	Zm00001d045675
	*ZmAsnS2*	GRMZM2G093175	Zm00001d044608
	*ZmAsnS3*	GRMZM2G053669	Zm00001d028750
	*THP9*/*ZmAsnS4*	GRMZM2G078472	Zm00001d047736

Note. Gene ID_v3 refers to gene identifiers from the maize (*Zea mays* L.) B73 RefGen_v3 reference genome annotation, while Gene ID_v4 refers to gene identifiers from the updated B73 RefGen_v4 reference genome annotation. Both versions are derived from the same maize B73 reference genome but represent different iterations of gene model curation and improvement.

## Data Availability

All data generated or analyzed during this study are available within the article or upon request from the corresponding author.

## Supplementary Materials

The following supporting information can be downloaded at: https://www.mdpi.com/article/10.3390/plants15091387/s1, Table S1: Median expression levels across breeding eras, trend classification, and pairwise statistical comparisons for retained genes; Table S2: Relative expression values and statistical comparisons for the 10 breeding-era-responsive TFs during nitrate and ammonium resupply assays; Table S3: Nucleotide diversity (π) and Tajima’s D values for 50-kb non-overlapping windows across the *ZmSPL19* ±50 kb region in 137 Chinese maize inbred lines from different breeding periods; Table S4: List of primer sequences for RT–qPCR; Figure S1: Expression responses of the 10 breeding-era-responsive TFs to nitrate and ammonium resupply. (a,b) RT-qPCR analysis of the 10 candidate TFs using root samples collected from the same nitrate starvation/resupply experiment described in Figure 3e. Relative transcript abundance was measured at 0, 1, 4, 8, and 12 h after KNO_3_ resupply and is shown as both a heatmap (a) and line plot (b). (c,d) RT-qPCR analysis of the same 10 candidate TFs after NH_4_Cl resupply following nitrogen starvation. Root samples were collected at 0, 1, 3, 6, and 12 h after NH_4_Cl addition and are shown as both a heatmap (c) and line plot (d). For each gene, relative expression was normalized to the internal reference gene and calculated using the 2^−ΔΔCt^ method, with the corresponding 0-h sample set to 1. The heatmaps and line plots represent the same relative expression values. Statistical comparisons were performed relative to the 0-h sample, and the corresponding exact values, raw data summaries, and significance results are provided in Supplementary Table S2. Data are means ± s.d. of three biological replicates.

## Abbreviations

The following abbreviations are used in this manuscript:

NUE	Nitrogen Use Efficiency
TF	Transcription Factor
NLPs	NIN-like proteins
SPL	Squamosa Promoter Binding Like
SPAD	Soil–Plant Analysis Development
AMT	Ammonium Transporter
NRT	Nitrate Transporter
NR	Nitrate Reductase
NIR	Nitrite Reductase
GS	Glutamine Synthetase
GOGAT	Glutamate Synthase
AS	Asparagine Synthetase
IMGE	Haploid-Inducer-Mediated Genome Editing

## References

[1] Erenstein O., Jaleta M., Sonder K., Mottaleb K., Prasanna B.M. (2022). Global maize production, consumption and trade: Trends and R&D implications. Food Secur..

[2] Shiferaw B., Prasanna B.M., Hellin J., Bänziger M. (2011). Crops that feed the world 6. Past successes and future challenges to the role played by maize in global food security. Food Secur..

[3] Ciampitti I.A., Vyn T.J. (2014). Understanding global and historical nutrient use efficiencies for closing maize yield gaps. Agron. J..

[4] Leghari S.J., Wahocho N.A., Laghari G.M., HafeezLaghari A., MustafaBhabhan G., HussainTalpur K., Bhutto T.A., Wahocho S.A., Lashari A.A. (2016). Role of nitrogen for plant growth and development: A review. Adv. Environ. Biol..

[5] Ladha J.K., Tirol-Padre A., Reddy C.K., Cassman K.G., Verma S., Powlson D.S., van Kessel C., Richter D.d.B., Chakraborty D., Pathak H. (2016). Global nitrogen budgets in cereals: A 50-year assessment for maize, rice and wheat production systems. Sci. Rep..

[6] Menegat S., Ledo A., Tirado R. (2022). Greenhouse gas emissions from global production and use of nitrogen synthetic fertilisers in agriculture. Sci. Rep..

[7] Shcherbak I., Millar N., Robertson G.P. (2014). Global metaanalysis of the nonlinear response of soil nitrous oxide (N_2_O) emissions to fertilizer nitrogen. Proc. Natl. Acad. Sci. USA.

[8] Zhang X., Davidson E.A., Mauzerall D.L., Searchinger T.D., Dumas P., Shen Y. (2015). Managing nitrogen for sustainable development. Nature.

[9] Cao H., Liu Z., Guo J., Jia Z., Shi Y., Kang K., Peng W., Wang Z., Chen L., Neuhaeuser B. (2024). ZmNRT1.1B (ZmNPF6.6) determines nitrogen use efficiency via regulation of nitrate transport and signalling in maize. Plant Biotechnol. J..

[10] Wu Q., Xu J., Zhao Y., Wang Y., Zhou L., Ning L., Shabala S., Zhao H. (2024). Transcription factor ZmEREB97 regulates nitrate uptake in maize (*Zea mays*) roots. Plant Physiol..

[11] Martin A., Lee J., Kichey T., Gerentes D., Zivy M., Tatout C., Dubois F., Balliau T., Valot B., Davanture M. (2006). Two cytosolic glutamine synthetase isoforms of maize are specifically involved in the control of grain production. Plant Cell.

[12] Huang Y., Wang H., Zhu Y., Huang X., Li S., Wu X., Zhao Y., Bao Z., Qin L., Jin Y. (2022). *THP9* enhances seed protein content and nitrogen-use efficiency in maize. Nature.

[13] Jia G., Chen G., Zhang Z., Tian C., Wang Y., Luo J., Zhang K., Zhao X., Zhao X., Li Z. (2025). Ferredoxin-mediated mechanism for efficient nitrogen utilization in maize. Nat. Plants.

[14] Ge M., Wang Y., Liu Y., Jiang L., He B., Ning L., Du H., Lv Y., Zhou L., Lin F. (2020). The NIN-like protein 5 (ZmNLP5) transcription factor is involved in modulating the nitrogen response in maize. Plant J..

[15] Wang R., Zhong Y., Han J., Huang L., Wang Y., Shi X., Li M., Zhuang Y., Ren W., Liu X. (2024). NIN-LIKE PROTEIN3.2 inhibits repressor *Aux*/*IAA14* expression and enhances root biomass in maize seedlings under low nitrogen. Plant Cell.

[16] Gu L., Cao Q., Dong J., Qiao M., Wang Z., Zhang Z., Sun H., Xie H., Ge M., Zhang Y. (2025). Transcription factor ZmNLP8 modulates nitrate utilization by transactivating *ZmNiR1.2* in maize. Plant J..

[17] Li C., Li Y., Song G., Yang D., Xia Z., Sun C., Zhao Y., Hou M., Zhang M., Qi Z. (2023). Gene expression and expression quantitative trait loci analyses uncover natural variations underlying the improvement of important agronomic traits during modern maize breeding. Plant J..

[18] Schaefer R.J., Michno J.-M., Jeffers J., Hoekenga O., Dilkes B., Baxter I., Myers C.L. (2018). Integrating coexpression networks with GWAS to prioritize causal genes in maize. Plant Cell.

[19] Shanks C.M., Rothkegel K., Brooks M.D., Cheng C.-Y., Alvarez J.M., Ruffel S., Krouk G., Gutiérrez R.A., Coruzzi G.M. (2024). Nitrogen sensing and regulatory networks: It’s about time and space. Plant Cell.

[20] Huang J., Cheng C.-Y., Brooks M.D., Jeffers T.L., Doner N.M., Shih H.-J., Frangos S., Katari M.S., Coruzzi G.M. (2025). Model-to-crop conserved NUE Regulons enhance machine learning predictions of nitrogen use efficiency. Plant Cell.

[21] Fei X., Wang Y., Zheng Y., Shen X., E L., Ding J., Lai J., Song W., Zhao H. (2022). Identification of two new QTLs of maize (*Zea mays* L.) underlying kernel row number using the HNAU-NAM1 population. BMC Genom..

[22] Wei H., Zhao Y., Xie Y., Wang H. (2018). Exploiting *SPL* genes to improve maize plant architecture tailored for high-density planting. J. Exp. Bot..

[23] Chen Q., Liu Z., Wang B., Wang X., Lai J., Tian F. (2015). Transcriptome sequencing reveals the roles of transcription factors in modulating genotype by nitrogen interaction in maize. Plant Cell Rep..

[24] Yang L., Guo S., Chen Q., Chen F., Yuan L., Mi G. (2016). Use of the stable nitrogen isotope to reveal the source-sink regulation of nitrogen uptake and remobilization during grain filling phase in maize. PLoS ONE.

[25] Zhao B., Xu M., Zhao Y., Li Y., Xu H., Li C., Kong D., Xie Y., Zheng Z., Wang B. (2022). Overexpression of *ZmSPL12* confers enhanced lodging resistance through transcriptional regulation of *D1* in maize. Plant Biotechnol. J..

[26] Wang J.L., Zhao X.Q., Zhang H.Q., Shen R.F. (2021). The preference of maize plants for nitrate improves fertilizer N recovery efficiency in an acid soil partially because of alleviated Al toxicity. J. Soils Sediments.

[27] Zhang H.-Q., Shen R.-F., Zhao X.-Q. (2022). Nitrogen source preference in maize at seedling stage Is mainly dependent on growth medium pH. Agronomy.

[28] Ren H., Liu Z., Wang X., Zhou W., Zhou B., Zhao M., Li C. (2025). Long-term excessive nitrogen application decreases spring maize nitrogen use efficiency via suppressing root physiological characteristics. J. Integr. Agric. (JIA).

[29] Xu C., Huang S., Tian B., Ren J., Meng Q., Wang P. (2017). Manipulating planting density and nitrogen fertilizer application to improve yield and reduce environmental impact in Chinese maize production. Front. Plant Sci..

[30] Fernandez J.A., Nippert J.B., Prasad P.V.V., Messina C.D., Ciampitti I.A. (2022). Post-silking ^15^N labelling reveals an enhanced nitrogen allocation to leaves in modern maize (*Zea mays*) genotypes. J. Plant Physiol..

[31] Haegele J.W., Cook K.A., Nichols D.M., Below F.E. (2013). Changes in nitrogen use traits associated with genetic improvement for grain yield of maize hybrids released in different decades. Crop Sci..

[32] Khosravi P., Gazestani V.H., Pirhaji L., Law B., Sadeghi M., Goliaei B., Bader G.D. (2015). Inferring interaction type in gene regulatory networks using co-expression data. Algorithms Mol. Biol..

[33] Wang B., Zhu L., Zhao B., Zhao Y., Xie Y., Zheng Z., Li Y., Sun J., Wang H. (2019). Development of a Haploid-Inducer Mediated Genome Editing System for Accelerating Maize Breeding. Mol. Plant.

[34] Li C., Liu C., Qi X., Wu Y., Fei X., Mao L., Cheng B., Li X., Xie C. (2017). RNA-guided Cas9 as an in vivo desired-target mutator in maize. Plant Biotechnol. J..

