# Supplementary material for: A Breeding-Informed Regulatory Screen Identifies ZmSPL19 as a Negative Regulator of Nitrogen-Sufficient Growth in Maize (Zea mays L.)
Source: Plants (Basel). 2026 Apr 30;15(9):1387. doi: 10.3390/plants15091387 (PMC13164645; doi:10.3390/plants15091387)
Supplement: Supplementary file 1 [file plants-15-01387-s001.zip › Supplementary Figure S1.pdf]

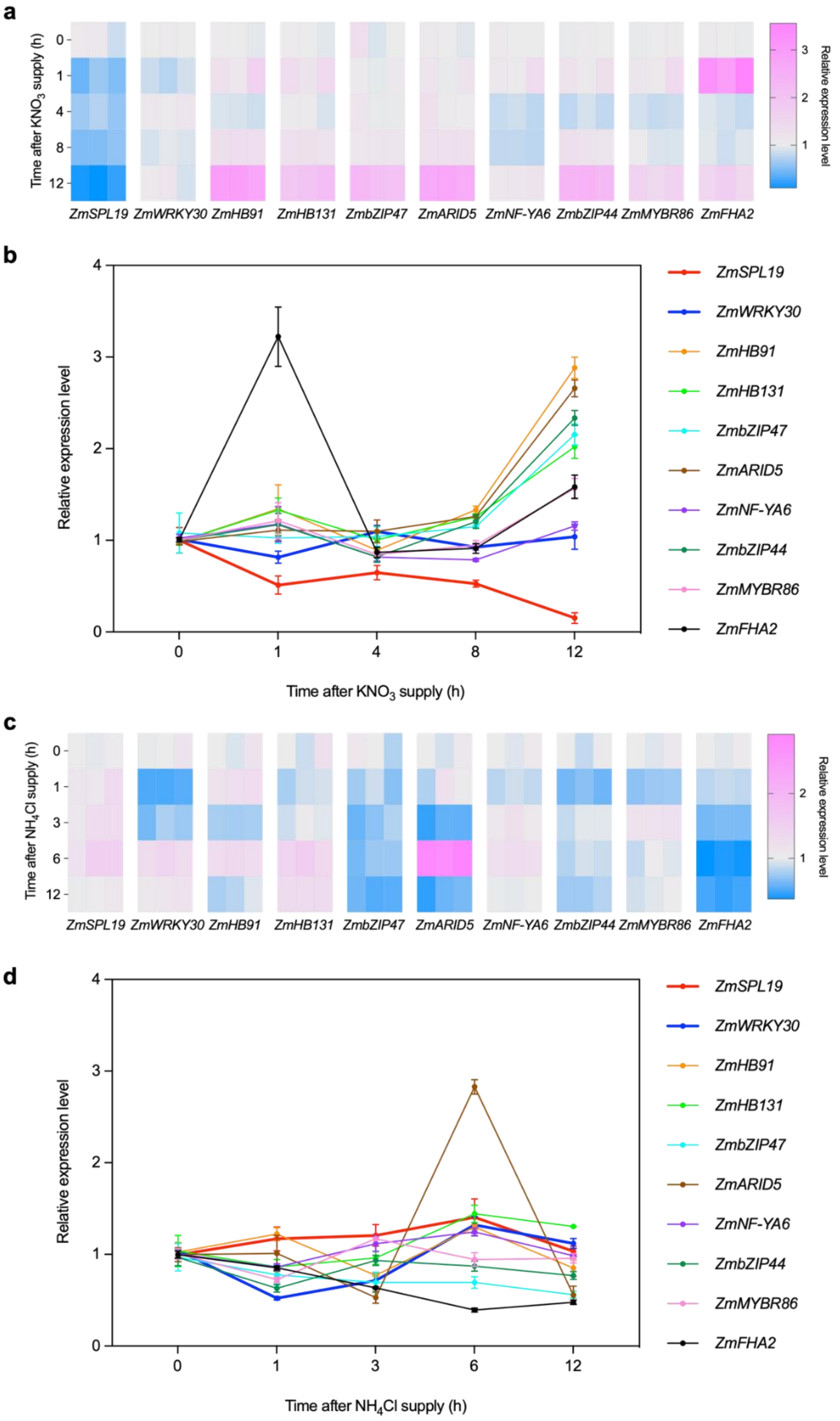

Figure S1. Expression responses of the 10 breeding-era-responsive TFs to nitrate and ammonium resupply. (**a,b**) RT-qPCR analysis of the 10 candidate TFs using root samples collected from the same nitrate starvation/resupply experiment described in Fig. 3e. Relative transcript abundance was measured at 0, 1, 4, 8, and 12 h after KNO_3 resupply and is shown as both a heatmap (**a**) and line

plot (**b**). (**c,d**) RT-qPCR analysis of the same 10 candidate TFs after NH_4Cl resupply following nitrogen starvation. Root samples were collected at 0, 1, 3, 6, and 12 h after NH_4Cl addition and are shown as both a heatmap (**c**) and line plot (**d**). For each gene, relative expression was normalized to the internal reference gene and calculated using the $2^{-\Delta\Delta Ct}$ method, with the corresponding 0-h sample set to 1. The heatmaps and line plots represent the same relative expression values. Statistical comparisons were performed relative to the 0-h sample, and the corresponding exact values, raw data summaries, and significance results are provided in Supplementary Table S3. Data are means ± s.d. of three biological replicates.
